# A microbiota-dependent bile acid reprograms alveolar macrophages to control lung inflammation

**DOI:** 10.1038/s41392-025-02552-w

**Published:** 2026-01-17

**Authors:** Magdalena Wolska, Pilar Rodríguez-Viso, Anna Świątkowska, Edyta Bulanda, Tomasz P. Wypych

**Affiliations:** Laboratory of Host-Microbiota Interactions, Nencki Institute of Experimental Biology, Polish Academy of Sciences, Warsaw, Poland

**Keywords:** Innate immunity, Translational immunology

**Dear Editor**,

The human microbiota orchestrates immune responses through both local cell-cell interactions and circulating metabolites. While microbiota-derived metabolites are particularly relevant at sites distal to the intestine, most have been described to act locally in the gut, with only a few, such as short-chain fatty acids or aromatic amino acid derivatives, shown to exert systemic effects.^1^ In this context, bile acids stand out for their potential to expand this space: although primarily shown to promote Treg cells and inhibit Th17 responses in the gut,^2^ microbial transformations generate a structurally diverse pool of bile acid derivatives, providing a rich platform for discovering compounds capable of shaping immunity at distal sites.^2^

To investigate how bile acids regulate immune response of the airways, we profiled a panel of bile acids along primary-to-secondary transformation pathways for their ability to modulate inflammatory responses in primary mouse lung immune cells stimulated with lipopolysaccharide (LPS) (Fig. 1a). This approach pointed to a single molecule, isolithocholic acid (isoLCA), as the most prominent immunomodulator in the lung. We next sought to pinpoint lung immune cell subsets responsive to isoLCA. Using fluorescence-activated cell sorting, we isolated alveolar macrophages, dendritic cells, monocytes/interstitial macrophages, and a remaining population from the lungs of naïve mice (Fig. 1a, upper right). Alveolar macrophages emerged as the primary producers of CXCL10 upon LPS stimulation, with isoLCA significantly reducing its production (Fig.1a, upper right). The effect was evolutionarily conserved in human monocyte-derived macrophages (MDMs) differentiated with M-CSF and IL-10, a condition previously shown to induce a transcriptional program resembling alveolar macrophages^3^ (Fig. 1a, bottom right).

**Fig. 1:**
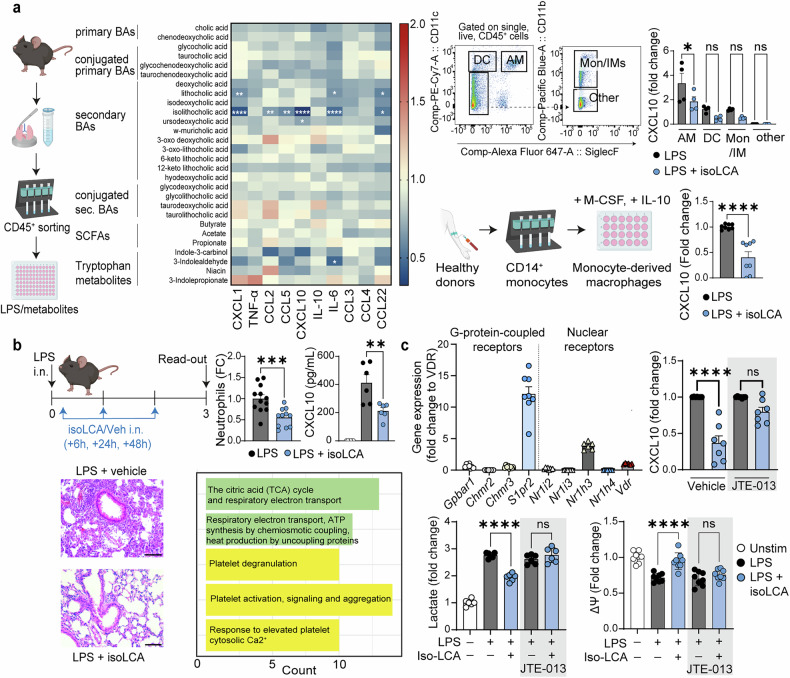
Microbiota-dependent bile acid, isolithocholic acid, targets lung immunity. **a** *Left*: Mouse primary lung immune cells (CD45^+^) were obtained *via* magnetic cell sorting and stimulated with LPS in the presence of tested metabolites (at 50 μM). The pro-inflammatory response was assessed 18 hours later by cytometric bead arrays and compared to control conditions (LPS + vehicle). Pooled data from four independent experiments, each performed in technical duplicates. *Upper Right*: Gating strategy for fluorescence-activated cell sorting (FACS): alveolar macrophages (AM) were sorted as CD11c^+^ SiglecF^+^, dendritic cells (DC) as CD11c^+^ SiglecF^-^, monocytes/interstitial macrophages (Mon/IMs) as CD11c^-^ SiglecF^-^ CD11b^+^, and other cells as CD11c^-^ SiglecF^-^ CD11b^-^. Cells were stimulated with LPS as per CD45^+^ cells. Unstimulated cells were used as the baseline for fold-change calculations. Data pooled from two independent experiments, each performed in technical duplicates. *Lower Right*: Human monocyte-derived macrophages (MDMs) were differentiated from CD14^+^ monocytes obtained from healthy donors using a cocktail of M-CSF and IL-10 and treated with LPS with or without isoLCA as before. Concentration of CXCL10 in culture supernatants was assessed 18 hours later. Data pooled from four independent experiments, each performed in technical duplicates. **b** Mice were administered with LPS intranasally on day 0, followed by intranasal instillation of isoLCA 6 h, 24 h, and 48 h later as per experimental setup. Influx of neutrophils into the lungs, concentration of CXCL10 in the BALF, hematoxylin and eosin (H&E) staining of lung sections (Scale bar = 100 μm) and transcriptional changes in alveolar macrophages were analyzed 72 hours after LPS administration. Data are pooled from three independent experiments (neutrophils, n = 12 mice for LPS+vehicle, n = 11 mice for LPS+isoLCA) and two independent experiments (CXCL10, n = 6 mice per group). The pathway analysis plot depicts the top five pathways induced by isoLCA treatment in alveolar macrophages. **c** *Top*: Expression of bile acid receptors in human MDMs. Data pooled from 3-4 donors, each qPCR performed in technical duplicates. CXCL10 production by human MDMs treated with LPS and isoLCA in the presence of S1PR2 antagonist, JTE-013. Data pooled from three independent experiments, two performed in technical duplicates and one in triplicates. *Bottom*: Secretion of lactate by human MDMs 18 hours post-treatment with LPS ± isoLCA in the presence/absence of the S1PR2 antagonist. Data pooled from three independent experiments, each performed in technical duplicates. Mitochondrial membrane potential of human MDMs assessed 18 hours post-treatment with LPS ± isoLCA in the presence/absence of the S1PR2 antagonist. Data pooled from three independent experiments, two performed in technical triplicates and one in duplicates. Statistical significance for the heatmap was determined with Two-Way analysis of variance (ANOVA) with Šidák correction for multiple comparisons. Statistical significance for graphs with two groups was evaluated using an unpaired Student’s t-test (for Gaussian distribution) or Mann-Whitney test (for non-Gaussian distribution). For graphs with more than two groups, statistical significance was determined using One-Way ANOVA with Šidák correction for multiple comparisons. Data distribution was assessed with the D’Agostino & Pearson normality test. Data are represented as mean ± SEM. *p ≤ 0.05, **p ≤ 0.01, ***p ≤ 0.001, ****p ≤ 0.0001. A figure includes elements created with BioRender

In vivo, intranasal isoLCA treatment reduced neutrophil influx, CXCL10 levels in bronchoalveolar lavage fluid (BALF), and inflammatory infiltrates in a mouse model of acute lung injury (Fig. 1b). Single-cell RNA sequencing of lung cells revealed transcriptional changes in alveolar macrophages, including upregulation of tricarboxylic acid (TCA) cycle genes, highlighting isoLCA-induced metabolic reprogramming in those cells (Fig. 1b). Notably, anti-inflammatory phenotype of MDM cells was abrogated under hypoxia or pharmacological TCA cycle inhibition, indicating a direct mechanistic link between metabolic rewiring and isoLCA’s immunomodulation. These results were validated in primary mouse lung immune cells (supplementary figure deposited at Figshare: 10.6084/m9.figshare.30920774).

To identify the receptor mediating isoLCA’s effects, we profiled bile acid receptor expression by qPCR in human MDMs. Among the candidates, sphingosine-1-phosphate receptor 2 (S1PR2) showed the highest expression (Fig. 1c). Pharmacological inhibition with the selective antagonist JTE-013 abolished isoLCA-mediated suppression of CXCL10, normalized lactate production, and prevented rescue of mitochondrial membrane potential, establishing S1PR2 as the mediator of isoLCA signaling (Fig. 1c).

Expanding to the model of respiratory infection, isoLCA treatment resulted in reduced neutrophil infiltration into the BALF. Consistent with isoLCA’s ability to suppress lactate production in macrophages, we noted a significant reduction in BALF lactate concentrations in treated mice. Also, we observed reduced levels of albumin, indicative of improved integrity of the epithelial barrier in the airways. In the mediastinal lymph nodes, we observed reduced numbers of CD4^+^ T cells and B cells, indicating that isoLCA can modulate not only innate but also adaptive immune responses. Finally, in plasma, we noted reduced levels of total IgG1 (supplementary figure deposited at Figshare: 10.6084/m9.figshare.30836510).

Collectively, our study uncovers a previously unrecognized role for the microbiota-dependent bile acid as a modulator of airway immunity through a novel mechanism. While secondary bile acids, including LCA and its derivatives, 3-oxo-LCA and isoLCA, have been shown to modulate intestinal CD4⁺ T cell differentiation *via* Vitamin D receptor or RORγt,^2^ our findings reveal a fundamentally different immune context and mechanistic axis: the cellular target is alveolar macrophages, the receptor is S1PR2, and the mechanism of action is metabolic reprogramming. This discovery broadens the scope of bile acid-mediated immunoregulation to include pulmonary innate immunity.

Our work also provides the missing link between clinical correlation and experimental validation. Previously, Stutz et al., observed reduced levels of secondary bile acids in COVID-19 patients who died from respiratory failure, suggesting that secondary bile acids might modulate respiratory immunity.^4^ Here, we provide experimental evidence supporting this hypothesis and identify the bile acid species with the most pronounced effects.

Finally, our data highlight metabolic reprogramming of alveolar macrophages as a therapeutic avenue in lung disease. This aligns with the work of Auger et al., who proposed metabolic rewiring as a mechanism underlying the anti-inflammatory effects of glucocorticosteroids, and suggested the untapped potential of small molecules that enhance the TCA cycle as candidates for a new class of anti-inflammatory drugs.^5^ Our findings reinforce this concept and position microbiota-dependent bile acids as promising candidates within this emerging therapeutic category.

In sum, our findings establish microbiota-dependent bile acids as a previously untapped reservoir of systemic immunometabolic modulators. Beyond the immediate translational implications for respiratory conditions, where intranasal isoLCA demonstrates protective activity, this work positions bile acid structural diversity as a discovery platform for novel immunometabolic regulators with relevance both within and beyond the gut.

## Supplementary information

Supplementary Material for "A microbiota-dependent bile acid reprograms alveolar macrophages to control lung inflammation".

## Data Availability

Raw scRNA-seq data are available at GEO: GSE307734, supplementary figures and remaining datasets at Figshare (10.6084/m9.figshare.30920774, 10.6084/m9.figshare.30836510, 10.6084/m9.figshare.30597152, 10.6084/m9.figshare.30919061).

## References

[1] Elinav, E. et al. Microbes and metabolites in immunity. *Immunity* **57**, 1995–1999 (2024). 39260349 10.1016/j.immuni.2024.08.011

[2] Mohanty, I. et al. The changing metabolic landscape of bile acids - keys to metabolism and immune regulation. *Nat. Rev. Gastroenterol. Hepatol.* **21**, 493–516 (2024). 38575682 10.1038/s41575-024-00914-3 PMC12248421

[3] Hoepel, W. et al. High titers and low fucosylation of early human anti-SARS-CoV-2 IgG promote inflammation by alveolar macrophages. *Sci. Transl. Med.* **13**, 1–16 (2021). 10.1126/scitranslmed.abf8654 PMC8158960 33979301

[4] Stutz, M. R. et al. Immunomodulatory fecal metabolites are associated with mortality in COVID-19 patients with respiratory failure. *Nat. Commun.* **13**, 1–11 (2022). 36329015 10.1038/s41467-022-34260-2 PMC9633022

[5] Auger, J. P. et al. Metabolic rewiring promotes anti-inflammatory effects of glucocorticoids. *Nature* **629**, 184–192 (2024). 38600378 10.1038/s41586-024-07282-7

