# Supplementary material for: A microbiota-dependent bile acid reprograms alveolar macrophages to control lung inflammation
Source: Signal Transduct Target Ther. 2026 Jan 17;11:35. doi: 10.1038/s41392-025-02552-w (PMC12812161; doi:10.1038/s41392-025-02552-w)
Supplement: Supplementary file 1 — Supplementary Material for "A microbiota-dependent bile acid reprograms alveolar macrophages to control lung inflammation". [file 41392_2025_2552_MOESM1_ESM.docx]

**Supplementary Material for**

**A microbiota-dependent bile acid reprograms alveolar macrophages to control lung inflammation**

Magdalena Wolska#, Pilar Rodríguez-Viso#, Anna Świątkowska, Edyta Bulanda, Tomasz P. Wypych^1^*

Laboratory of Host-Microbiota Interactions, Nencki Institute of Experimental Biology, Polish Academy of Sciences, Warsaw, Poland

# equal contribution

*Correspondence to: [t.wypych@nencki.edu.pl](mailto:t.wypych@nencki.edu.pl) (T.P.W)

**This file includes:**

Materials and Methods

**Experimental animals**

Wild-type C57BL/6J mice were obtained from the Nencki Institute of Experimental Biology, Warsaw, Poland, or the Experimental Medicine Centre of the Medical University of Bialystok, Bialystok, Poland. All mice were kept under controlled conditions (12h light/dark cycle, 22±1˚C, 75±5% humidity), maintained under specific-pathogen-free conditions and fed *ad libitum* with irradiated regular chow diet and distilled water. 8-12 weeks old male and female mice were used for all experiments. All animal experiments were approved by the 1^st^ Local Ethical Committee for Experiments on Animals (Permissions nr 1255/2022, 1573/2024).

**Mouse model of acute lung injury**

Mice were anesthetized by inhalation of 3.5% vetflurane (Virbac) in oxygen for 6 min. Lipopolysaccharide (LPS) from *Escherichia coli* O127:B8 (Merck) was reconstituted in sterile phosphate-buffered saline (PBS) (Life Technologies). 20 µg of LPS in a volume of 30 µL was administered intranasally on day 0. Mice were humanely euthanized with a lethal dose of pentobarbital (200 mg/kg, Biowet) on day 3.

**Isolithocholic acid treatment *in vivo***

Mice were anesthetized by inhalation of 3.5% vetflurane in oxygen for 6 min. Isolithocholic acid (Cayman) was reconstituted in DMSO at 50 mM and further diluted in sterile PBS so that mice received the dose of 200 µg/Kg body weight by intranasal application of 40 μL of the solution at the time points indicated in the figures. Control mice received 40 µL of sterile PBS with appropriate supplementation with DMSO.

**Bronchoalveolar lavage fluid (BALF) collection**

BALF was collected by flushing airways with 0.5 mL PBS supplemented with 0.1% BSA (Merck). After centrifugation at 1200 *×g* for 10 min at 4˚C, the cellular pellet was collected for FACS analysis, and the supernatant was stored at -20˚C until further use.

**ELISA**

Concentrations of CXCL10 in culture supernatants were measured with the Mouse DuoSet ELISA kits (R&D) or the Human DuoSet ELISA (R&D) according to the manufacturer's instructions. Concentrations of IFN-γ, TNF-α, CCL2 (MCP-1), IL-12p70, CCL5 (RANTES), GM-CSF, IL-10, IFN-β, and IFN-α were measured with LEGENDplex™ Mouse Proinflammatory Chemokine Panel (BioLegend) and LEGENDplex™ Mouse Anti-Virus Response Panel (BioLegend) according to the manufacturer's instructions.

**Flow cytometry**

Lungs were finely cut with scissors and digested with collagenase IV (Gibco^TM^) in RPMI 1640 medium (Life Technologies) for 50 min at 37˚C in gentle shaking (350 rpm), filtered through a 40 µm cell strainer (Biologix), and washed with PBS supplemented with 1% FBS and 2 mM EDTA (Eurx) (named here as the “FACS buffer”). Erythrocytes were lysed using a concentrated ammonium chloride buffer (150 mM NH_4_Cl + 1M Tris-HCl, pH 7.4). Mediastinal lymph nodes were filtered through 40 µm cell strainer and washed as the lung cells. Cells were stained with a mix of antibodies in FACS buffer for 20 min at 4˚C in a 96-well round-bottom plate (GenoPlast Biotech S.A.). Alveolar macrophages (AMs) and dendritic cells (DCs) were identified using antibodies against CD11c-PE/Cy7 (BioLegend, 117318, diluted 1:200 in FACS buffer) and SiglecF-AF647 (BD Biosciences, 562680, 1:200). Neutrophils were identified using antibodies against CD11b-PB (BioLegend, 101224, 1:1600) and Ly6G-BUV395 (BD Biosciences, 563978, 1:200). Inflammatory monocytes were identified using antibodies against CD11b-PB and Ly6C-PE (BioLegend, 128008, 1:1600). T helper cells were identified using antibodies against CD3-AF700 (BioLegend, 100216, 1:200) and CD4-FITC (BioLegend, 100510, 1:200). Cells were acquired on a BD Fortessa (BD Biosciences) with fluorescence-activated cell sorting (FACS) BD FACS DiVA^TM^ v6.3.1 software and analyzed with FlowJo v10.10.0 software (Tree Star).

**Histology**

Left lung lobes were fixed in 10 mL of 10% buffered formalin (Alpinus Chemia) at 4˚C. Next, the tissues were dehydrated and embedded in paraffin. To remove water and prepare the tissues for paraffin embedding, a tissue dehydration scheme was followed, and the tissues were incubated sequentially in 70% ethanol for 15 min, then in 96% ethanol for 15 min, and then twice in 100% ethanol, each time for 30 min. After the dehydration step, the tissues were transferred to xylene (twice for 20 minutes each), which allowed the transition from alcohol to hydrophobic paraffin. Paraffin impregnation was carried out at 60°C, twice for 1 hour each, using paraffin with a melting point of 56-58°C. All process steps were carried out using an automatic tissue processor (e.g., Thermo Scientific, Tissue Processor STP-120). After the process was completed, the lungs were embedded in paraffin blocks using a Thermo Scientific Histostar apparatus. The tissues were oriented to obtain cross sections through the lung lobe. The finished blocks were left to solidify on a cold plate (about 4°C) and then stored at room temperature until further processing. Microtomy was performed using a rotary microtome (e.g., Thermo Scientific, Microm HE 340E). The paraffin block was trimmed and cut into 5 µm-thick slices. Paraffin ribbons were transferred to a water bath at 40-45°C to expand and then applied to Superfrost Plus slides. The slides were dried overnight at 52°C to fix the sections on the medium. Staining of the slides was carried out using the hematoxylin and eosin (H&E) method. Sections were deparaffinized in xylene (twice for 5 minutes each), then rehydrated in an alcoholic series of decreasing concentrations (ethanol: 100%, 96%, 70%) and rinsed in distilled water. The sections were then stained with Harris hematoxylin (Leica, Surgipath Harris Haematoxylin 3801562E) for 10 minutes, washed with tap water and differentiated in 70% alcohol acidified with 1% HCl for 30 seconds. For nuclear staining, a “blueing” process was performed in Scott's Tap Water Substitute (Leica, Surgipath Scott's Tap Water Substitute, 3802901E). After staining with 1% aqueous eosin (Leica, Surgipath Aqueous Eosin 1%, 3801592E) for 3 minutes, the slides were dehydrated in 96% and 100% ethanol, then cleaned in xylene and mounted using resin mounting medium (Thermo Scientific, Shandon Consul-Mount 9990440).

**Stimulation of lung CD45^+^ cells**

C57BL/6J mice aged 8-12 weeks were sacrificed with a lethal dose of pentobarbital, and 0.75 mL of dispase II (8 mg mL^-1^, Roche) was injected intratracheally, followed by intratracheal injection of 0.5 mL 1% low-melting agarose (Sigma-Aldrich). Lungs were then covered with ice for 5 min, removed, and placed in a 15 mL Falcon tube with 2 mL of dispase II and incubated for 1h at RT with gentle agitation (350 RPM). Then, the lungs were mechanically disrupted using forceps in DMEM supplemented with DNAse I (1 U mL^-1^; Sigma-Aldrich). Lung homogenates were first filtered through 70 µm and subsequently 40 µm cell strainers (Biologix), and centrifuged for 10 min, 300 *xg* at 4˚C. Erythrocytes were lysed with RBC lysis solution for 15 min at 4˚C. Cells were then labeled with anti-CD45-bio, anti-CD16/32-bio, and anti-Ter119-bio (Biolike), followed by incubation with streptavidin microbeads and sorted using LS columns on a MACS sorter into CD45^+^ and CD45^-^ cells. CD45^+^ cells were plated in 96-well round-bottom plates at a density of 2 x 10^5^ per well and stimulated with LPS (10 ng mL^-1^; Merck) for 18h at 37 ˚C and 5% CO_2_, after which the cell supernatants were collected and stored at -20 ˚C until further use.

**Human monocyte-derived macrophage cultures**

Permission to obtain peripheral blood from healthy donors was obtained from the Ethical Committee of the Warsaw Medical University (permission nr KB/125). Peripheral blood was collected from healthy donors, and blood mononuclear cells (PBMCs) were isolated using Lymphoprep density gradient medium (STEMCell^TM^) at 800*×g* for 30 min. Mononuclear cells were washed, and erythrocytes were lysed using RBC lysis buffer for 15 min at RT. Then, CD14^+^ monocytes were sorted using anti-CD14-biotin (Biolike) and streptavidin microbeads (Biolike) in a QuadroMACS separator (Miltenyi Biotec). Purified monocytes were plated in RPMI supplemented with 1% FBS and 1% penicillin/streptomycin for 2 h to let them attach. Then, attached monocytes were cultured in RPMI supplemented with 10% FBS and 1% penicillin/streptomycin, containing 50 ng mL^-1^ human recombinant M-CSF (BioLike) for 6 days. At day 7, media was replaced by RPMI supplemented with 10% FBS and 1% penicillin/streptomycin, containing 50 ng mL^-1^ recombinant human IL-10 (Bio-Techne) for 1 day^1, 2^. Obtained human monocyte-derived macrophages (MDMs) were detached with cell scrapers (NEST) and cultured in 96-well flat-bottom plates (Genos) at a density of 7 x 10^4^ cells per well and pre-treated with isoLCA (50 µM) for 2 h before stimulation with LPS (100 ng mL^-1^) for 18h at 37 ˚C and 5% CO_2_. When indicated, cells were pre-treated with 1 µM of JTE-013 antagonist (Merck) for 2 h before treatment with isoLCA.

**Isolation of RNA, complementary DNA (cDNA) synthesis and qRT-PCR**

TRIzol™ was added to cell pellets, and RNA was extracted with the Total RNA Zol-Out™ kit (A&A Biotechnology) according to manufacturer instructions. RNA was quantified in a DS-11 series Spectrophotometer (DeNovix), and first-strand complementary DNA (cDNA) was obtained from 100 ng of total RNA, using the High Capacity cDNA Reverse Transcription kit (Life Technologies). qRT-PCR was performed using the CFX Opus 384 Real-Time PCR System (Bio-Rad). Reactions were carried out in a 10 µL final volume containing 5 µL of SYBR® Green Fast Advanced Cells-to-CT kit (Applied Biosystems^TM^), 4.5 µL of cDNA in nuclease-free water (GenoPlast), and 0.5 µL of each forward and reverse primer (8 µM, Bio-Rad or Genomed). PCR conditions were 95˚C for 2 min, 40 cycles of 5 s denaturation at 95˚C, 30 s annealing at 60˚C, followed by 30 s elongation at 60˚C. Primers for genes of interest or housekeeping genes are deposited at 10.6084/m9.figshare.30919061. Data were analysed using the ΔΔC_T_ method.

**Determination of mitochondrial membrane potential**

MDM cells were cultured in black 96-well flat transparent bottom plates (Corning) at a density of 7 × 10^4^ per well, and pre-treated with isoLCA (50 µM) for 2 h before stimulation with LPS (100 ng mL^-1^) for 18 h. After stimulation, cells were washed with pre-warmed PBS containing CaCl_2_ (WARCHEM) and MgCl_2_ (WARCHEM), followed by incubation with Tetramethylrhodamine methyl ester perchlorate (TMRM, 50-250 nM, Sigma-Aldrich) for 30 min at 37˚C to determine the mitochondrial membrane potential. After incubation, cells were washed with PBS containing CaCl_2_ and MgCl_2_. Fluorescence was determined (excitation: 548 nm, emission: 574 nm) using a TECAN microplate reader.

**L-Lactate production**

MDM cells were cultured in 96-well flat-bottom plates at a density of 7 × 10^4^ per well in complete media without FBS. Cells were pre-treated with isoLCA (50 µM) for 2 h before stimulation with LPS [100 ng mL^-1^ ] for 18 h. L-lactate was detected in culture supernatants after stimulation using the Lactate-Glo™ kit (Promega) following the manufacturer's instructions.

**Single-cell RNA sequencing and analysis**

CD45^+^ and CD45^-^ lung cells were isolated from mice as described above, fixed according to the manufacturer’s instructions (ScaleBio Low Volume Fixation), and stored at -80˚C. Library preparation of single cell suspension derived from mouse lungs was done using ScaleBio’s (San Diego, California) single-cell technology, which uses a combinatorial indexing strategy to target single cells. Cells coming from 12 samples (4 conditions) were distributed to 12 wells of the first Index RT 96-well plate. Cells were transferred through a series of barcoded plates (96-well index RT plate, 384 index ligation plate, and final 96-well indexed PCR plate) using ScaleBio’s patented Split&Pool method, which uniquely labels the cDNA of each cell in each well of the plate and allows it to go through a standard RNAseq library preparation. Cells were enzymatically digested prior to PCR amplification, and once amplification and purification were complete, the libraries were ready for sequencing. Libraries were sequenced with the NGS sequencing platform for short reads (Nova Seq 6000, Illumina San Diego, California). The final concentration of the obtained library and its purity, expressed as A260/280 values (range of normal parameter values 1.8-2.2), was measured using the Nanodrop device (Thermo Scientific, Waltham, Massachusetts), as well as using the Quantus Fluorometer and its dedicated QuantiFluor dsDNA System reagent kit (Promega, Madison, Wisconsin, USA). The quality of the library was assessed using the Agilent 2100 Bioanalyzer and its dedicated Agilent High Sensitivity DNA Kit (Agilent, Santa Clara, California). Sample demultiplexing, alignment to the reference genome (GRCm39), and calculating count matrices were performed by the ScaleRNA pipeline v.1.3.2 (https://github.com/ScaleBio/ScaleRna). The default parameters were used, apart from the minCellRatio parameter, which was set to 100. The library structure used was the libV1.

The secondary analysis was performed in R v. 4.2.2 (R Core Team (2022). R: A language and environment for statistical computing. R Foundation for Statistical Computing, Vienna, Austria. URL https://www.R-project.org/.), using the Seurat v. 5.0.2 package^3^. Only the cells with at least 200, but no more than 10000 unique feature counts were considered in the analysis. The cells with more than 5% of mitochondrial features were filtered out. The data was first normalized using the NormalizeData function with the normalization method set as „LogNormalize”, then scaled using the ScaleData function using all features, then PCA was run, using variable features of all samples to determine the dimensionality of the dataset, which was set at 15. The data was then integrated using the IntegrateLayer function using the CCAIntegration method. To cluster the cells, a set of FindNeighbors and FindClusters functions were used, with a maximum of 15 dimensions and a 0.4 resolution set, respectively. Next, the RunUMAP function was used to reduce the dimensionality of the data. ScType (https://academic.oup.com/bioinformatics/article/40/7/btae426/7700663) and singleR^4^ packages were used to aid in annotating cell clusters. The cluster markers were identified using the FindAllMarkers function. The differentially expressed genes were identified using the FindMarkers function using the Wilcoxon Rank Sum test. The functional analysis was performed using the enrichPathway function from the ReactomePA package (v. 1.42.0). P-value < 0.05 was considered significant. Plots were generated using the ggplot2 (v. 3.3.6) and enrichplot (v. 1.18.4) (https://github.com/WXlab-NJMU/scrna-recom/tree/main) packages.

1. Chen, H.J. *et al.* Meta-Analysis of in vitro-Differentiated Macrophages Identifies Transcriptomic Signatures That Classify Disease Macrophages in vivo. *Front Immunol* **10**, 2887 (2019).

2. Hoepel, W. *et al.* High titers and low fucosylation of early human anti-SARS-CoV-2 IgG promote inflammation by alveolar macrophages. *Sci Transl Med* **13** (2021).

3. Nader, K. *et al.* ScType enables fast and accurate cell type identification from spatial transcriptomics data. *Bioinformatics* **40** (2024).

4. Aran, D. *et al.* Reference-based analysis of lung single-cell sequencing reveals a transitional profibrotic macrophage. *Nat Immunol* **20**, 163-172 (2019).
